# Characteristics of patients with COVID-19 who have deteriorating chest X-ray findings within 48 h: a retrospective cohort study

**DOI:** 10.1038/s41598-023-49340-6

**Published:** 2023-12-12

**Authors:** Tatsuya Kusumoto, Shotaro Chubachi, Ho Namkoong, Hiromu Tanaka, Ho Lee, Shiro Otake, Kensuke Nakagawara, Takahiro Fukushima, Atsuho Morita, Mayuko Watase, Takanori Asakura, Katsunori Masaki, Hirofumi Kamata, Makoto Ishii, Naoki Hasegawa, Norihiro Harada, Tetsuya Ueda, Soichiro Ueda, Takashi Ishiguro, Ken Arimura, Fukuki Saito, Takashi Yoshiyama, Yasushi Nakano, Yoshikazu Mutoh, Yusuke Suzuki, Ryuya Edahiro, Koji Murakami, Yasunori Sato, Yukinori Okada, Ryuji Koike, Yuko Kitagawa, Katsushi Tokunaga, Akinori Kimura, Seiya Imoto, Satoru Miyano, Seishi Ogawa, Takanori Kanai, Koichi Fukunaga

**Affiliations:** 1 Division of Pulmonary Medicine, Department of Medicine, Keio University School of Medicine, 35 Shinanomachi, Shinjuku-ku, Tokyo, 160-8582 Japan; 2 Department of Infectious Diseases, Keio University School of Medicine, 35 Shinanomachi, Shinjuku-ku, Tokyo, 160-8582 Japan; 3 Department of Respiratory Medicine, National Hospital Organization Tokyo Medical Center, Tokyo, Japan; 4 Department of Pulmonary Medicine, Saitama City Hospital, Saitama, Japan; 5 Department of Respiratory Medicine, Juntendo University Faculty of Medicine and Graduate School of Medicine, Tokyo, Japan; 6 Department of Respiratory Medicine, Osaka Saiseikai Nakatsu Hospital, Osaka, Japan; 7 Department of Internal Medicine, Japan Community Health Care Organization (JCHO), Saitama Medical Center, Saitama, Japan; 8 Department of Respiratory Medicine, Saitama Cardiovascular and Respiratory Center, Kumagaya, Japan; 9 Department of Respiratory Medicine, Tokyo Women’s Medical University, Tokyo, Japan; 10 Department of Emergency and Critical Care Medicine, Kansai Medical University General Medical Center, Moriguchi, Japan; 11 Respiratory Disease Center, Fukujuji Hospital, Japan Anti-Tuberculosis Association, Tokyo, Japan; 12 Department of Internal Medicine, Kawasaki Municipal Ida Hospital, Kawasaki, Japan; 13 Department of Infectious Diseases, Tosei General Hospital, Seto, Japan; 14 Department of Respiratory Medicine, Kitasato University Kitasato Institute Hospital, Tokyo, Japan; 15 Department of Statistical Genetics, Osaka University Graduate School of Medicine, Suita, Japan; 16 Department of Respiratory Medicine, Tohoku University Graduate School of Medicine, Sendai, Japan; 17 Department of Preventive Medicine and Public Health, Keio University School of Medicine, Tokyo, Japan; 18 Integrated Frontier Research for Medical Science Division, Institute for Open and Transdisciplinary Research Initiatives, Osaka University, Suita, Japan; 19 The Center for Infectious Disease Education and Research (CiDER), Osaka University, Suita, Japan; 20 Laboratory of Statistical Immunology, Immunology Frontier Research Center (WPI-IFReC), Osaka University, Suita, Japan; 21 Medical Innovation Promotion Center, Tokyo Medical and Dental University, Tokyo, Japan; 22 Department of Surgery, Keio University School of Medicine, Tokyo, Japan; 23 Genome Medical Science Project (Toyama), National Center for Global Health and Medicine, Tokyo, Japan; 24 Institute of Research, Tokyo Medical and Dental University, Tokyo, Japan; 25 Division of Health Medical Intelligence, Human Genome Center, The Institute of Medical Science, The University of Tokyo, Tokyo, Japan; 26 M&D Data Science Center, Tokyo Medical and Dental University, Tokyo, Japan; 27 Department of Pathology and Tumor Biology, Kyoto University, Kyoto, Japan; 28 Institute for the Advanced Study of Human Biology (WPI-ASHBi), Kyoto University, Kyoto, Japan; 29 Department of Medicine, Center for Hematology and Regenerative Medicine, Karolinska Institute, Stockholm, Sweden; 30 Division of Gastroenterology and Hepatology, Department of Medicine, Keio University School of Medicine, Tokyo, Japan

**Keywords:** Epidemiology, Outcomes research, Translational research

## Abstract

The severity of chest X-ray (CXR) findings is a prognostic factor in patients with coronavirus disease 2019 (COVID-19). We investigated the clinical and genetic characteristics and prognosis of patients with worsening CXR findings during early hospitalization. We retrospectively included 1656 consecutive Japanese patients with COVID-19 recruited through the Japan COVID-19 Task Force. Rapid deterioration of CXR findings was defined as increased pulmonary infiltrates in ≥ 50% of the lung fields within 48 h of admission. Rapid deterioration of CXR findings was an independent risk factor for death, most severe illness, tracheal intubation, and intensive care unit admission. The presence of consolidation on CXR, comorbid cardiovascular and chronic obstructive pulmonary diseases, high body temperature, and increased serum aspartate aminotransferase, potassium, and C-reactive protein levels were independent risk factors for rapid deterioration of CXR findings. Risk variant at the *ABO* locus (rs529565-C) was associated with rapid deterioration of CXR findings in all patients. This study revealed the clinical features, genetic features, and risk factors associated with rapid deterioration of CXR findings, a poor prognostic factor in patients with COVID-19.

## Introduction

The pandemic of coronavirus disease (COVID-19) caused by the severe acute respiratory syndrome coronavirus 2 (SARS-CoV-2) has resulted in high morbidity and mortality rates worldwide. The disease rapidly spread throughout the world; as of December 29, 2022, there were 663,380,366 confirmed cases and 6,691,567 deaths worldwide^1^. COVID-19 severity varies according to several factors, including patient characteristics^2–5^ and laboratory findings^6–8^. Furthermore, the severity of chest X-ray (CXR) findings in patients with COVID-19 can predict various outcomes, including duration of hospitalization^9,10^, use of invasive mechanical ventilation (IMV)^11–13^, intensive care unit (ICU) admission^11–13^, and mortality^14–18^.

Chest imaging facilitates the diagnosis and management of patients^19^. In clinical settings, CXR can be easily performed using mobile CXR units in a dedicated, isolated room to reduce the transmission risk^19^. Although CXR is considered less sensitive for detecting pulmonary involvement in early-stage disease^20^, it is useful for monitoring the progression of lung abnormalities in COVID-19, especially in critically ill ICU patients^21^. The requirement for ventilatory support is associated with worsening findings early after admission^22^; moreover, mortality can be predicted based on CXR findings before and after ICU admission^23^. However, the impact of deteriorating CXR findings on outcomes other than ventilatory support needs and the clinical and genetic characteristics of patients with deteriorating CXR findings remain unclear.

A nationwide multi-center consortium was established to address the COVID-19 pandemic in Japan^24,25^. Since the pandemic's onset, the network has been collecting DNA, RNA, and plasma samples, as well as detailed clinical information, from patients with COVID-19 throughout Japan on a long-term basis. The first Japanese large-scale genome-wide association study (GWAS) on COVID-19 reported that genetic variants, including *DOCK2*, had a population-specific association with oxygenation requirements by patients with COVID-19^24^.

It is important for clinicians to be able to predict the severity of a patient's illness, especially during a pandemic, in order to use medical resources appropriately. We hypothesised that rapid deterioration in CXR findings after hospitalisation is associated with subsequent worsening of the disease. We aimed to investigate the prognosis, clinical characteristics, and genetic characteristics of patients with worsening CXR findings during early hospitalization.

## Methods

### Study design and settings

**Figure 1:**
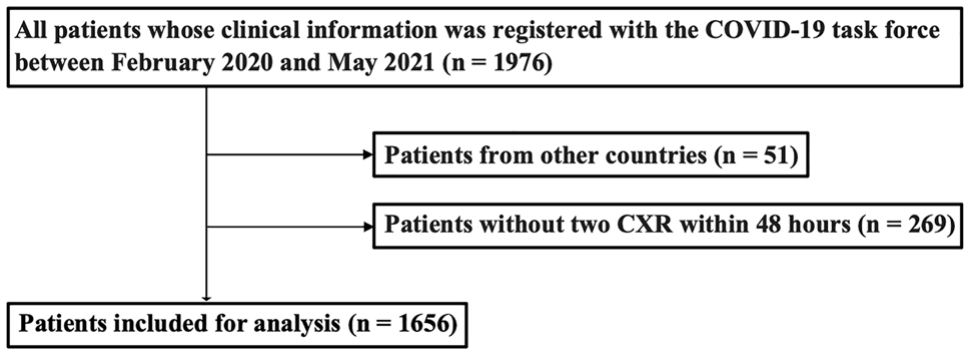
Participant selection process Overall, 1976 patients with COVID-19 were hospitalized during the study period. We excluded 51 non-Japanese patients and 269 patients with incomplete medical records. COVID-19, coronavirus disease.

This retrospective cohort study recruited hospitalized COVID-19 cases through the Japan COVID-19 Task Force^24^. From February 2020 to May 2021, data obtained from consecutive patients aged ≥ 18 years, who were diagnosed with COVID-19 based on polymerase chain reaction tests and agreed to participate in the study, were entered into electronic case record forms by attending physicians at the affiliated research institutions. The exclusion criteria were as follows: (1) patients from other countries (2) patients with incomplete medical records, e.g., insufficient data regarding the presence/absence of chest radiographic deterioration within 48 h of admission or critical outcomes (Fig. 1). All patients provided written or oral informed consent. The study design was approved by the ethics committees of Keio University School of Medicine (20,200,061) and related research institutions. All methods were performed in accordance with the relevant guidelines and regulations.

### Data collection

The following characteristics were extracted from each electronic case record form: age, sex, height, weight, clinical symptoms and signs, laboratory findings on admission, comorbidities, and disease severity (ICU admission, IMV usage, and survival status). All laboratory tests were performed based on the patient’s clinical care needs. The recorded symptoms and signs included those observed at the times of referral and admission and during hospitalization. Laboratory and radiographic findings were collected within 48 h of the initial visit or admission. Rapid deterioration of CXR findings was defined as increased lung infiltrates in > 50% of the lung fields within 48 h compared with those at admission, which is based on the criteria for severe disease in patients with COVID-19^26^. One attending physician at each facility reviewed and assessed the initial and follow-up CXRs. The timing of CXR imaging was at the discretion of the attending physician. The collected data were reviewed by a team of respiratory clinicians. Missing core data were obtained by contacting the clinician. Missing background data were noted as unknown. We defined disease severity as follows: most severe: patients requiring high-flow oxygen device support, invasive mechanical ventilation, extracorporeal membrane oxygenation, or death; severe: patients requiring low-flow oxygen device support; mild: symptomatic patients not requiring oxygen support; and asymptomatic: asymptomatic patients not requiring oxygen support^25^.

### Genotype characteristics of the patients with COVID-19

We performed GWAS genotyping of 2520 patients with COVID-19 using the Infinium Asian Screening Array (Illumina). We applied stringent quality control (QC) filters to the samples and variants. A total of 2393 COVID-19 cases passed the sample QC (details described elsewhere^24^) and underwent genome-wide genotype imputation (details described elsewhere^24^). Among them, 1,169 had information regarding chest radiographs. Among 18 known COVID-19-related risk variants, we evaluated the effects of 15 risk variants with imputation scores of > 0.7 on the rapid deterioration of CXR findings (See Supplementary Table 1 in the Supplementary Material)^24,27–35^.

### Statistical analysis

Regarding baseline characteristics, categorical variables were presented as frequencies and proportions, while continuous variables were presented as means and standard deviations. We compared data according to the presence/absence of lung infiltrates in > 50% of the fields within 48 h of admission using the t-test and Chi-square test, as appropriate. The median hospitalization duration was estimated using the Kaplan–Meier method and compared using the log-rank test.

To investigate the association between rapid deterioration of imaging and radiographic findings, CXR findings (unilateral/bilateral ground-glass opacity [GGO]/consolidation) were adjusted, followed by multivariate logistic regression analysis. Additionally, a Cochran–Armitage trend test was performed to determine the rapid deterioration of CXR findings as well as the tendency of radiographic findings to exhibit no, unilateral, or bilateral shadows.

To assess the association between the rapid deterioration of CXR findings and clinical outcomes (death, most severe disease, IMV use, and ICU treatment), we performed multivariate logistic regression analyses with adjustment for baseline CXR findings, number of days from symptom onset to hospitalisation, and characteristics known as predictors of COVID-19 severity (age, body mass index [BMI], hypertension, diabetes mellitus, cardiovascular disease, malignancy, chronic obstructive pulmonary disease [COPD], asthma, chronic liver disease, and chronic kidney disease)^2–5^.

To identify the clinical characteristics of patients with rapid deterioration of CXR findings, we adopted a holdout method, where data from two-thirds of the cases were used as training data, while the remaining data were used as test data to validate the performance of the prediction model for patients with COVID-19 who have rapid deterioration of CXR findings. Receiver operating characteristic (ROC) curve analysis was performed to determine appropriate cut-off values of continuous variables for rapid deterioration of CXR findings using the Youden index. We performed multivariable analysis using a logistic regression model with a backward selection procedure to select the combinations of variables. The variables were selected based on a threshold *p*-value of 0.05. The Bayesian information criterion was also applied to select the optimal model among the existing models. We performed ROC curve analysis for the model, with the performance of the prediction models being assessed using the area under the curve (AUC).

The dosage effects of the variants on rapid deterioration of CXR findings were evaluated using logistic regression models, with age (included only in all age analyses) and sex as covariates.

To evaluate the association between the ABO blood groups and rapid deterioration of CXR findings, we performed a multivariate logistic regression analysis of the A/B/AB/O blood groups and other blood groups, with adjustment for age and sex.

To ensure the reliability of the data, two clinicians from different affiliations, M.W. (National Hospital Organization Tokyo Medical Center) and T.K. (Keio University School of Medicine), independently reviewed the initial and follow-up CXRs and assessed for rapid deterioration of CXR findings (n = 45). Agreement between the two clinicians’ readings was analysed using Cohen's kappa coefficient (κ), with κ values graded as slight (0.00–0.20), fair (0.21–0.40), moderate (0.41–0.60), substantial (0.61–0.80), and almost perfect (0.81–1.00) according to Landis and Koch criteria^36^.

Data were presented as adjusted odds ratios (aORs) with 95% confidence intervals (CIs). Statistical significance was set at *p* < 0.05. All statistical analyses were performed using the JMP 16 program (SAS Institute) and SAS software (version 9.4; SAS Institute).

### Statement of Ethics

All patients involved in this study provided written or oral informed consent, and the study design was approved by the ethics committees of Keio University School of Medicine (20,200,061) and the affiliated research institutions.

## Results

### Patient baseline characteristics

Table 1 summarizes the baseline characteristics of the patients (n = 1656; 538 women). Comparison of two clinicians’ ratings of rapid deterioration of CXR findings showed almost perfect agreement, with a Cohen's kappa coefficient (κ) of 0.85 (95% CI 0.55–1.00). Among them, 168 (10.1%) patients experienced rapid deterioration of CXR findings; further, these patients were generally older, had a higher BMI, and had a longer interval between symptom onset and hospitalization (all *P* < 0.001). Rapid deterioration of CXR findings were associated with a higher rate of comorbidities, including hypertension (*P* = 0.044), diabetes mellitus (*P* < 0.001), cardiovascular disease (*P* = 0.017), COPD (*P* < 0.001), and chronic kidney disease (*P* = 0.002), as well as a higher frequency of symptoms, including fever (*P* < 0.001), cough (*P* < 0.001), sputum (*P* = 0.006), rhinorrhea (*P* = 0.029), shortness of breath (*P* < 0.001), abdominal pain (*P* = 0.022), diarrhea (*P* = 0.036), nausea (*P* < 0.001), and fatigue (*P* < 0.001). We analyzed patient characteristics according to baseline CXR findings; we found similar trends in the presence/absence of imaging findings and rapid deterioration of CXR findings (Supplementary Tables 2).

**Table 1:** Baseline characteristics of the patients.

Parameters	All patients	Patients showing no deterioration of CXR findings	Patients showing rapid deterioration of CXR findings	*P* value
	(n = 1656)	(n = 1488, 89.9%)	(n = 168, 10.1%)	
Demographics				
Age, years	59.5 (± 17.6)	59.0 (± 18.0)	64.2 (± 12.9)	< 0.001
Sex, female/male (%)	32.5/67.5	32.9/67.1	28.6/71.4	0.253
BMI, kg/m^2^	24.6 (± 4.8)	24.4 (± 4.9)	26.0 (± 4.4)	< 0.001
Current or previous smoker (%)	46.8	46.2	51.9	0.175
Brinkman index	660.8 (± 802.7)	645.0 (± 758.1)	781.1 (± 1087.7)	0.165
Number of days from symptom onset to hospitalisation, days	6.0 (± 4.6)	5.8 (± 4.3)	7.2 (± 6.5)	< 0.001
Comorbidities				
Hypertension (%)	37.1	36.2	44.2	0.044
Diabetes mellitus (%)	23.3	21.9	35.7	< 0.001
Cardiovascular disease (%)	10.8	10.2	16.3	0.017
Malignancy (%)	6.9	6.8	7.2	0.851
Autoimmune disease (%)	3.7	3.9	2.4	0.340
COPD (%)	5.0	4.4	10.2	< 0.001
Asthma (%)	6.8	6.9	6.1	0.690
Hyperuricemia (%)	11.1	10.8	14.3	0.167
Chronic liver disease (%)	3.3	3.5	1.9	0.266
Chronic kidney disease (%)	8.0	7.4	14.3	0.002
Signs and symptoms				
Unconsciousness (%)	3.8	3.7	4.9	0.442
Fever (≥ 37.5℃) (%)	78.6	77.4	89	< 0.001
Cough (%)	58.8	57.3	71.3	< 0.001
Sputum (%)	24.3	23.3	32.9	0.006
Sore throat (%)	23.1	22.9	24.1	0.735
Rhinorrhea (%)	14.5	13.9	20.3	0.029
Dysgeusia (%)	16.6	17	13.3	0.218
Olfactory disorder (%)	14.3	14.8	10.3	0.122
Shortness of breath (%)	32.6	30.4	52.1	< 0.001
Abdominal pain (%)	2.7	2.4	5.4	0.022
Abdominal distension (%)	0.7	0.6	1.8	0.090
Hematochezia (%)	0.4	0.5	0.0	0.372
Diarrhea (%)	16.2	15.6	22.0	0.036
Nausea (%)	8.3	7.3	17.5	< 0.001
Fatigue (%)	48.9	47	65.9	< 0.001

Data are shown as mean ± standard deviation or percentage values. Data were analyzed using the χ^2^ test, t-test, or log-rank test, as appropriate.

BMI, body mass index; CXR, chest X-ray; COPD, chronic obstructive pulmonary disease.

Compared with patients with two CXRs within 48 h, those without were generally younger (*P* < 0.001) and comprised more women (*P* = 0.001), and patients with less severity (*P* = 0.003) (Supplementary Table 3).

Supplementary Table 4 summarizes the clinical features of the patients. The vital signs of patients with rapid deterioration of CXR findings were characterized by increased body temperature (*P* < 0.001), heart rate (*P* < 0.001), respiratory rate (*P* < 0.001), decreased SpO_2_ (*P* < 0.001), and a high rate of a requirement for oxygen support (*P* < 0.001). Further, their blood test findings included increased white blood cell counts (*P* = 0.0477) and neutrophil ratios (*P* < 0.001) as well as increased blood urea nitrogen (*P* < 0.001), creatinine (*P* = 0.006), lactate dehydrogenase (*P* < 0.001), brain natriuretic peptide (*P* = 0.018), ferritin (*P* < 0.001), hemoglobin A1c (*P* < 0.001), fibrinogen (*P* < 0.001), D-dimer (*P* = 0.003), and C-reactive protein (CRP) (*P* < 0.001) levels. Moreover, they presented with decreased lymphocyte ratios (*P* < 0.001), platelet counts (*P* < 0.001), albumin (*P* < 0.001), and sodium (*P* < 0.001) levels.

### Association between baseline CXR findings and rapid deterioration

**Figure 2:**
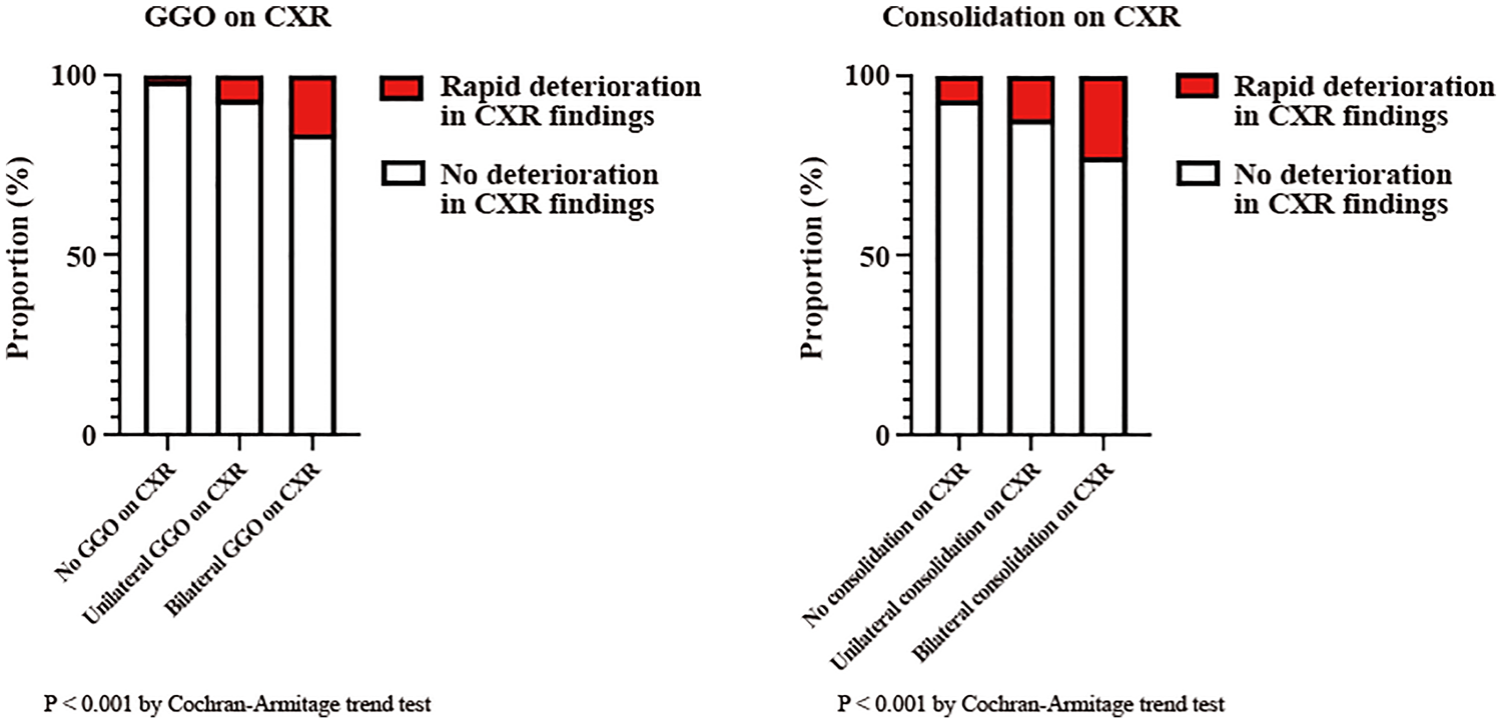
Radiographic findings in patients with rapid deterioration of CXR findings. Proportion of cases showing rapid deterioration of CXR findings according to the distribution of GGO/consolidation on CXR. CXR, chest X-ray; GGO, ground-glass opacity.

Figure 2 shows the comparison of the baseline CXR findings between patients with and without rapid deterioration of CXR findings. For patients with GGO and consolidation on both modalities, the frequency of rapid deterioration of CXR findings increased in the following order: no shadow, unilateral shadow, and bilateral shadow; CXR GGO (*P* < 0.001), CXR consolidation (*P* < 0.001). Multivariate logistic regression analysis revealed that unilateral shadow, bilateral shadow, GGO, and consolidation were associated with an increased likelihood of rapid deterioration of CXR findings (Table Supplementary Table 5).

### Association between rapid deterioration of CXR findings and outcomes

Table 2 shows the results of multiple logistic regression analysis using parameters that included rapid deterioration of CXR findings, GGO, consolidation on CXR, and number of days from symptom onset to hospitalisation, in addition to the previously reported prognostic factors^2–5^. Rapid deterioration of CXR findings was an independent risk factor for death (aOR [95% CI] = 3.12 [1.45–6.74]), most severe disease (aOR [95% CI] = 3.03 [1.98–4.62]), use of IMV (aOR [95% CI] = 1.96 [1.24–3.09]), and ICU treatment (aOR [95% CI] = 2.33 [1.60–3.41]).

**Table 2:** Predictors of death, most severe disease, IMV use, and ICU treatment.

Variable	Death			Most severe disease			IMV use			ICU treatment		
	aOR	95% CI	*P* value	aOR	95% CI	*P* value	aOR	95% CI	*P* value	aOR	95% CI	*P* value
Rapid deterioration of CXR findings	3.12	1.45–6.74	0.004	3.03	1.98–4.62	< 0.001	1.96	1.24–3.09	0.002	2.33	1.60–3.41	< 0.001
CXR GGO	2.65	0.84–8.40	0.097	3.11	1.72–5.61	< 0.001	2.61	1.39–4.93	0.003	1.70	1.21–2.39	0.002
CXR consolidation	2.83	1.38–5.81	0.005	2.75	1.94–3.89	< 0.001	2.94	2.02–4.29	< 0.001	1.27	0.95–1.69	0.103
Age, years	1.08	1.05–1.12	< 0.001	1.03	1.02–1.05	< 0.001	1.02	1.01–1.04	0.004	1.01	0.99–1.02	0.266
BMI, kg/m^2^	1.01	0.93–1.10	0.774	1.05	1.01–1.09	0.022	1.06	1.01–1.10	0.010	1.03	0.99–1.06	0.075
Number of days from symptom onset to hospitalisation, days	0.97	0.90–1.05	0.497	1.06	1.03–1.10	< 0.001	1.09	1.05–1.13	< 0.001	1.02	0.99–1.05	0.163
Hypertension	1.11	0.54–2.28	0.781	1.33	0.92–1.92	0.128	1.39	0.94–2.07	0.101	0.93	0.69–1.25	0.632
Diabetes mellitus	0.96	0.46–2.01	0.914	1.83	1.27–2.65	0.001	1.86	1.25–2.76	0.002	1.35	0.99–1.83	0.058
Cardiovascular disease	1.97	0.89–4.35	0.092	1.27	0.77–2.08	0.346	1.29	0.76–2.18	0.350	1.15	0.76–1.74	0.521
Malignancy	0.82	0.23–2.96	0.759	0.71	0.35–1.47	0.361	0.70	0.31–1.58	0.396	1.45	0.88–2.37	0.146
COPD	0.57	0.15–2.24	0.422	1.74	0.92–3.28	0.088	1.87	0.96–3.65	0.065	1.02	0.58–1.80	0.950
Asthma	2.07	0.63–6.82	0.231	0.78	0.37–1.64	0.515	0.67	0.29–1.53	0.337	1.15	0.69–1.92	0.580
Chronic liver disease	2.32	0.47–11.5	0.303	1.50	0.63–3.58	0.357	1.57	0.63–3.91	0.335	1.13	0.58–2.22	0.723
Chronic kidney disease	3.95	1.81–8.62	< 0.001	2.12	1.28–3.49	0.003	1.88	1.10–3.21	0.021	1.61	1.03–2.50	0.036

The adjusted odds ratio was estimated by logistic regression.

For continuous variables, the unit odds ratio for each 1-unit change is shown.

95% CI, 95% confidence interval; aOR, adjusted odds ratio; BMI, body mass index; COPD, chronic obstructive pulmonary disease; CXR, chest X-ray; GGO, ground-glass opacity; ICU, intensive care unit; IMV, invasive mechanical ventilation.

### Risk factors for rapid deterioration of CXR findings

**Figure 3:**
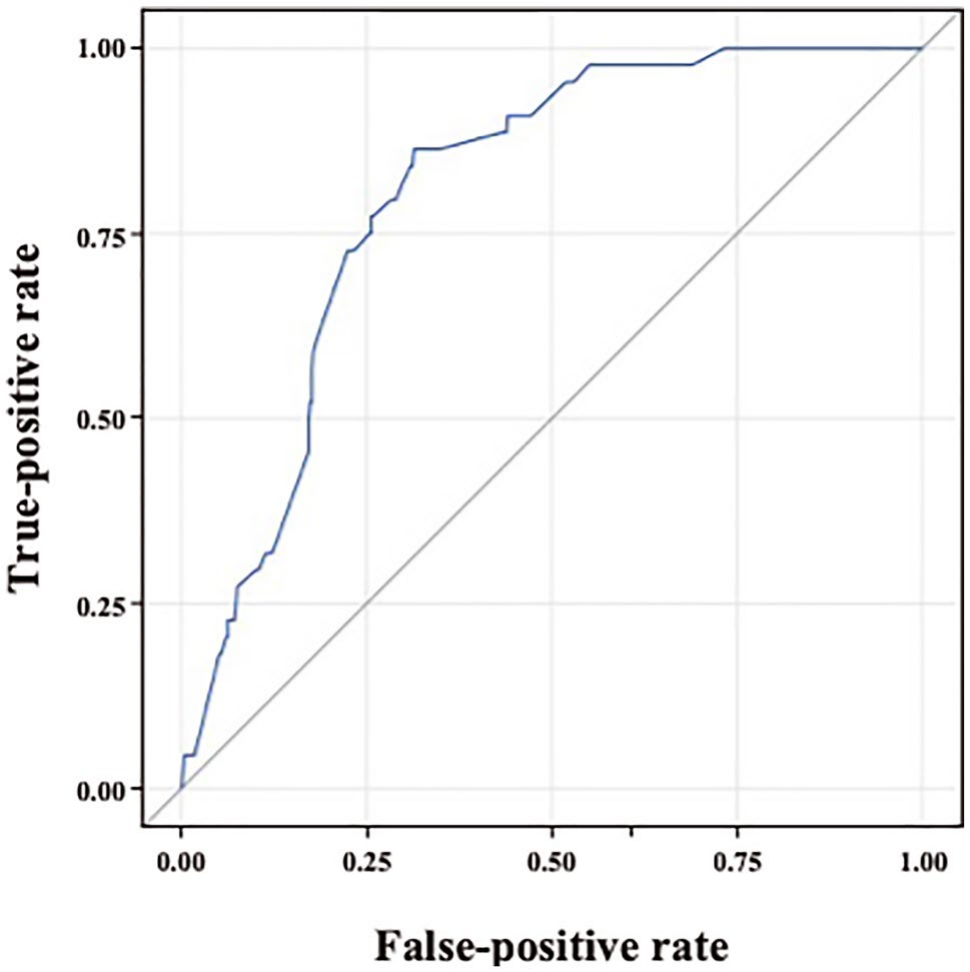
ROC curve of the multivariate logistic regression model for predicting rapid deterioration of CXR findings using CXR consolidation; comorbid cardiovascular disease; comorbid COPD; body temperature; and AST, K, and CRP levels in patients with COVID-19. Using test data, AUC = 0.806. ROC, receiver operating characteristic; CXR, chest X-ray; COPD, chronic obstructive pulmonary disease; AST, aspartate aminotransferase; K, potassium; CRP, C-reactive protein; COVID-19, coronavirus disease; AUC, area under the curve.

Table 3 displays the results of the logistic regression analysis performed to clarify the risk factors for rapid deterioration of CXR findings, and Fig. 3 shows the ROC curve. The presence of consolidation (bilateral consolidation (aOR [95% CI] = 3.07 [1.91–4.93]), unilateral consolidation (aOR [95% CI] = 2.19 [1.11–4.32]) on CXR, coexisting cardiovascular disease (aOR [95% CI] = 2.00 [1.12–3.58]), coexisting COPD (aOR [95% CI] = 2.32 [1.12–4.79]), body temperature ≥ 37.7 °C (aOR [95% CI] = 2.53 [1.64–3.91]), aspartate aminotransferase (AST) ≥ 30 IU/L (aOR [95% CI] = 2.31 [1.38–3.87]), serum potassium ≥ 4.3 mEq/L (aOR [95% CI] = 1.75 [1.11–2.78]), and CRP ≥ 2.53 mg/dL (aOR [95% CI] = 3.16 [1.74–5.73]) were independent risk factors for rapid deterioration of CXR findings. The AUC (95% CI) was estimated to be 0.806 (0.753–0.858).

**Table 3:** Predictors of rapid deterioration in imaging findings.

Endpoint	Variable	aOR	95% CI	*P* value
Rapid deterioration in CXR findings	CXR bilateral consolidation	3.07	1.91–4.93	< 0.001
	CXR unilateral consolidation	2.19	1.11–4.32	
	Cardiovascular disease	2.00	1.12–3.58	0.019
	COPD	2.32	1.12–4.79	0.023
	Body temperature ≥ 37.7 °C	2.53	1.64–3.91	< 0.001
	AST ≥ 30 IU/L	2.31	1.38–3.87	0.002
	K ≥ 4.3 mEq/L	1.75	1.11–2.78	0.017
	CRP ≥ 2.53 mg/dL	3.16	1.74–5.73	< 0.001

The adjusted odds ratio was estimated by logistic regression.

95% CI, 95% confidence interval; aOR, adjusted odds ratio; AST, aspartate aminotransferase; COPD, chronic obstructive pulmonary disease; CRP, C-reactive protein; CXR, chest X-ray; K, potassium.

### Association of COVID-19-associated risk variants with rapid deterioration of CXR findings

Table 4 shows the dosage effects of COVID-19-related risk variants on rapid deterioration of CXR findings. rs529565-C (*ABO*) was associated with rapid deterioration of CXR findings in patients of all ages (aOR [95% CI] = 1.67 [1.23–2.26]; *P* < 0.001) and those aged < 65 years (aOR [95% CI] = 2.00 [1.31–3.05]; *P* = 0.001).

**Table 4:** Association of COVID-19 risk variants with rapid deterioration in CXR findings.

Endpoint	rsID	Gene	RA	EA	All ages			Age < 65 years		
					aOR	95% CI	*P* value	aOR	95% CI	*P* value
Rapid deterioration in CXR findings	rs60200309	*DOCK2*	G	A	1.47	0.95–2.27	0.084	1.73	0.97–3.07	0.061
	rs1886814	*FOXP4*	A	C	1.31	0.95–1.79	0.094	1.09	0.70–1.69	0.711
	rs72711165	*TMEM65*	T	C	1.27	0.60–2.68	0.538	0.82	0.28–2.44	0.723
	rs6020298	*TMEM189-UBE2V1*	G	A	0.88	0.67–1.18	0.401	0.74	0.48–1.14	0.170
	rs529565	*ABO*	T	C	1.67	1.23–2.26	< 0.001	1.91	1.25–2.94	0.003
	rs77534576	*TAC4*	C	T	1.32	0.70–2.51	0.391	1.19	0.50–2.80	0.699
	rs2109069	*DPP9*	G	A	1.13	0.73–1.74	0.589	1.19	0.66–2.14	0.570
	rs13050728	*IFNAR2*	T	C	0.78	0.57–1.07	0.119	0.77	0.50–1.18	0.228
	rs12252	*IFITIM3*	A	G	1.01	0.72–1.42	0.969	1.50	0.92–2.45	0.108
	rs429358	*APOE*	T	C	1.37	0.88–2.15	0.168	1.65	0.92–2.95	0.092
	rs12329760	*TMPRSS2*	C	T	0.88	0.64–1.21	0.429	1.02	0.66–1.58	0.914
	rs2271616	*SLC6A20*	G	T	1.10	0.70–1.71	0.679	0.82	0.42–1.62	0.575
	rs10774671	*OAS1*	G	A	0.89	0.62–1.27	0.519	0.89	0.54–1.49	0.666
	rs4801778	*PLEKHA4*	G	T	1.20	0.50–2.90	0.681	2.18	0.60–7.99	0.238
	rs11919389	None	T	C	1.19	0.88–1.59	0.263	1.13	0.74–1.73	0.570

The adjusted odds ratio was estimated by logistic regression, with adjustment for age and sex.

The odds ratio represents the incremental odds for each unit increase in allele dosage.

The significance threshold based on Bonferroni’s correction was set at *p* < 0.0033.

95% CI, 95% confidence interval; aOR, adjusted odds ratio; EA, effect allele; RA, reference allele; COVID-19, coronavirus disease; CXR, chest X-ray.

### Association of the ABO blood groups with rapid deterioration of CXR findings

Table 5 shows the association between rapid deterioration of CXR findings and the ABO blood groups. Blood group AB was associated with an increased risk of rapid deterioration of CXR findings compared with the non-AB blood types (aOR [95% CI] = 1.84 [1.15–2.95]), after adjustment for age and sex.

**Table 5:** The association of the ABO blood group with rapid deterioration of CXR findings.

Endpoint	ABO blood group	aOR	95% CI	*P* value
Rapid deterioration of CXR findings	A versus AB/B/O	1.12	0.80–1.57	0.522
	B versus A/AB/O	0.87	0.57–1.31	0.494
	AB versus A/B/O	1.84	1.15–2.95	0.011
	O versus A/AB/B	0.67	0.44–1.02	0.065

The adjusted odds ratio was estimated by logistic regression with adjustments for age and sex.

95% CI, 95% confidence interval; aOR, adjusted odds ratio; CXR, chest X-ray.

## Discussion

Our study provided three novel findings with clinical relevance. First, patients with COVID-19 with rapid CXR deterioration had poorer clinical outcomes than those without; accordingly, and they may require more aggressive treatment. Second, we identified predictors of CXR deterioration upon admission. Therefore, clinicians should pay more attention to CXR deterioration after hospitalization of patients with these risk factors. Third, we identified the genetic risk factors for CXR deterioration in Japanese patients with COVID-19.

The CXR severity score on admission is a risk factor for death, severe disease, IMV use, and ICU treatment in patients with COVID-19^9,11,12,14,15^. Deterioration of CXR findings after hospitalization influences the requirement for ventilatory support after admission^22^. Moreover, studies have investigated mortality prediction using CXR findings before and after ICU admission^23^ and the association of the worst CXR scores during hospitalization with discharge and death^37^. However, we found that rapid deterioration of CXR findings was an independent risk factor for death, most severe disease, ICU admission, and tracheal intubation. Furthermore, multivariate analysis using baseline CXR findings revealed that these relationships were robust. Compared with baseline CXR findings, rapid deterioration of CXR findings had a higher aOR of predicting worse outcomes. Our findings suggest clinicians should be aware of CXR deterioration, especially within 48 h of admission, regardless of the baseline CXR findings.

We identified the risk factors for rapid CXR deterioration. Several reported factors contribute to severe COVID-19 development^2–8^. In our study, patients with rapid CXR deterioration showed many of these risk factors for death and severe disease. CXR consolidation; concomitant cardiovascular disease and COPD; and elevated body temperature, AST, serum potassium, and CRP levels, were independent risk factors for rapid deterioration of CXR findings. A prediction model for CXR deterioration using these risk factors showed high accuracy (AUC = 0.806). These results may facilitate the prediction of rapid deterioration of CXR findings and prompt interventions.

Previous GWAS reports have suggested an association between the genetic characteristics of patients with COVID-19 and the severity of COVID-19^24,38^. We performed analyses using risk variants and ABO blood groups to characterize the genetic characteristics related to rapid deterioration of CXR findings. We identified whole-population and population-specific risk variants at the *ABO* locus (rs529565-C) among the 15 genes extracted from previous reports^24,27–35^. The *DOCK2* locus has been associated with severe disease in patients with COVID-19 aged < 65 years^24^. Therefore, we performed an analysis of the association between DOCK2 and acute worsening of imaging in patients with COVID-19 aged < 65 years, but found no significant differences. Regarding the risk variant of the *ABO* locus, we observed a risk of rapid deterioration of CXR findings in patients with COVID-19 in the AB blood group. In patients with COVID-19, an association between the AB blood group and most severe disease has been reported^39^. The AB blood group is reported to be more susceptible to a variety of infections^40^ and at higher risk for thrombosis^41^, which may contribute to the same mechanisms as most severe disease and rapid deterioration of CXR findings. However, further studies are needed to explore this point.

Our study had three main limitations. First, we could not determine the mechanisms underlying rapid deterioration of CXR findings from a virological perspective. Therefore, our findings may not reflect the current clinical picture of COVID-19 since epidemics of different variants have become serious public health threats in Japan and other countries^42,43^. Second, since this was a multi-center study, the interpretation of radiograms may have differed among centers. Although previous studies have performed clinical investigations based on quantitative evaluation of CXR findings, such quantitative evaluations were unavailable in our study^22,37^. Specifically, although ‘deterioration of lung infiltrates in > 50% of the lung field’ is a qualitative parameter, it may involve an increased risk of ambiguity. However, one strength of this multi-center study was that we enrolled a larger sample size than previous CXR-related studies on patients with COVID-19. Moreover, our prediction model for clinical outcomes applied a very simple assessment index that can be easily used in daily clinical practice. One problem with the definition of rapid deterioration of CXR findings was that if the baseline CXR revealed a shadow more extensive than half of the lung field, it did not meet the definition, irrespective of the subsequent deterioration degree. Nonetheless, despite this bias, we still achieved significant results; moreover, we did not discuss the management of possible controversial cases. Third, we excluded 269 patients without two chest X-rays within 48 h. Clinically severe patients tended to undergo repeated CXRs; therefore, these missing data might have led to selection bias.

## Conclusions

Rapid deterioration of CXR findings within 2 days of admission is a significant prognostic risk factor in patients with COVID-19. Accordingly, carefully monitoring changes in radiographic findings during this period is important. Specifically, patients with CXR consolidation, comorbid cardiovascular disease or COPD, fever with high inflammatory response, and elevated AST or blood potassium levels should be carefully monitored after admission.

### Supplementary Information

Supplementary Tables.

## Data Availability

All data generated or analysed during this study are included in this published article and its supplementary information files.

## Abbreviations

CXR: Chest X-ray
COVID-19: Coronavirus disease
SARS-CoV-2: Severe acute respiratory syndrome coronavirus 2
ICU: Intensive care unit
GWAS: Genome-wide association study
IMV: Invasive mechanical ventilation
QC: Quality control
GGO: Ground-glass opacity
BMI: Body mass index
COPD: Chronic obstructive pulmonary disease
ROC: Receiver operating characteristic
AUC: Area under the curve
aOR: Adjusted odds ratio
CI: Confidence interval
AST: Aspartate aminotransferase
CRP: C-reactive protein
