# Supplementary material for: Characteristics of patients with COVID-19 who have deteriorating chest X-ray findings within 48 h: a retrospective cohort study
Source: Sci Rep. 2023 Dec 12;13:22054. doi: 10.1038/s41598-023-49340-6 (PMC10716517; doi:10.1038/s41598-023-49340-6)
Supplement: Supplementary file 1 — Supplementary Tables. [file 41598_2023_49340_MOESM1_ESM.docx]

| **Supplementary Table 1.** COVID-19-related risk variants | | | | | |
| --- | --- | --- | --- | --- | --- |
| rsID | Gene | Allele | Risk allele frequency | Rsq | References |
| rs60200309 | *DOCK2* | G/A | 0.10659 | 0.87703 | [22] |
| rs35081325 | *LZTFL1* | A/T | 0.00157 | 0.60609 | [22, 25] |
| rs1886814 | *FOXP4* | A/C | 0.28850 | 0.89285 | [22, 26] |
| rs72711165 | *TMEM65* | T/C | 0.03410 | 0.97854 | [22, 26] |
| rs6020298 | *TMEM189-UBE2V1* | G/A | 0.52891 | 0.97819 | [27] |
| rs529565 | *ABO* | T/C | 0.46169 | 0.99999 | [22, 28] |
| rs77534576 | *TAC4* | C/T | 0.04697 | 0.97345 | [22, 26] |
| rs2109069 | *DPP9* | G/A | 0.11731 | 0.98286 | [22, 26] |
| rs13050728 | *IFNAR2* | T/C | 0.44176 | 0.95298 | [22, 26] |
| rs12252 | *IFITIM3* | A/G | 0.57575 | 0.72585 | [29-31] |
| rs429358 | *APOE* | T/C | 0.09826 | 0.99329 | [32] |
| rs12329760 | *TMPRSS2* | C/T | 0.37826 | 0.93708 | [33] |
| rs2271616 | *SLC6A20* | G/T | 0.12670 | 0.89671 | [26] |
| rs10774671 | *OAS1* | G/A | 0.79128 | 0.93241 | [26] |
| rs1819040 | *KANSL1* | T/A | 0.00167 | 0.24736 | [26] |
| rs4801778 | *PLEKHA4* | G/T | 0.02480 | 0.95039 | [26] |
| rs74956615 | *FDX2* and *RAVER1* | T/A | 0.00253 | 0.24672 | [26] |
| rs11919389 | None | T/C | 0.37635 | 0.97894 | [26] |

| **Supplementary Table 2.** Association between baseline CXR findings and patient baseline characteristics | | | | | | | | |
| --- | --- | --- | --- | --- | --- | --- | --- | --- |
| Parameters | No GGO on CXR | Unilateral GGO on CXR | Bilateral GGO on CXR | P value | No consolidation on CXR | Unilateral consolidation on CXR | Bilateral consolidation on CXR | P value |
|  | (n = 501, 31.3%) | (n = 208, 13.0%) | (n = 890, 55.7%) |  | (n = 1109, 70.1%) | (n = 140, 8.9%) | (n = 332, 21.0%) |  |
| **Demographics** |  |  |  |  |  |  |  |  |
| Age, years | 52.8 (±20.5) | 61.3 (±16.7) | 63.8 (±14.1) | <0.001 | 58.4 (±18.2) | 63.4 (±17.3) | 64.4 (±13.7) | <0.001 |
| Sex, female/male (%) | 38.3/61.7 | 27.9/72.1 | 29.6/70.4 | 0.001 | 32.9/67.1 | 30.0/70.0 | 31.0/69.0 | 0.679 |
| BMI, kg/m^2^ | 23.9 (±4.8) | 24.0 (±4.6) | 25.3 (±4.9) | <0.001 | 24.7 (±4.8) | 23.9 (±4.6) | 25.0 (±5.2) | 0.124 |
| Current or previous smoker (%) | 42.4 | 40.9 | 51.4 | 0.001 | 46.6 | 49.2 | 47.2 | 0.850 |
| Brinkman index | 455.0 (±591.1) | 811.6 (±864.8) | 738.7 (±862.5) | <0.001 | 604.3 (±713.1) | 758.1 (±740.2) | 864.7 (±1086.1) | 0.004 |
| Number of days from symptom onset to hospitalisation, days | 4.5 (±5.0) | 5.3 (±3.2) | 6.9 (±4.3) | <0.001 | 5.4 (±4.2) | 6.0 (±3.6) | 7.7 (±5.4) | <0.001 |
| **Comorbidities** |  |  |  |  |  |  |  |  |
| Hypertension (%) | 28.8 | 37.8 | 42.6 | <0.001 | 34.9 | 36.8 | 47.5 | <0.001 |
| Diabetes mellitus (%) | 16.5 | 23.1 | 28.2 | <0.001 | 22.1 | 27.9 | 29.0 | 0.021 |
| Cardiovascular disease (%) | 10.9 | 12.0 | 11.0 | <0.001 | 11.1 | 13.6 | 10.4 | 0.595 |
| Malignancy (%) | 6.7 | 9.3 | 6.4 | 0.342 | 7.1 | 9.4 | 5.0 | 0.185 |
| Autoimmune disease (%) | 3.4 | 1.5 | 4.1 | 0.181 | 3.6 | 0.0 | 4.6 | 0.044 |
| COPD (%) | 3.0 | 6.9 | 5.8 | 0.038 | 4.3 | 7.3 | 7.0 | 0.069 |
| Asthma (%) | 5.8 | 7.9 | 7.0 | 0.546 | 6.8 | 3.6 | 7.4 | 0.304 |
| Hyperuricemia (%) | 8.9 | 10.2 | 12.6 | 0.100 | 12.0 | 10.7 | 8.0 | 0.127 |
| Chronic liver disease (%) | 3.1 | 3.9 | 3.6 | 0.814 | 3.8 | 2.3 | 3.1 | 0.617 |
| Chronic kidney disease (%) | 4.9 | 9.4 | 9.7 | 0.007 | 7.8 | 9.8 | 9.2 | 0.590 |
| **Signs and symptoms** |  |  |  |  |  |  |  |  |
| Unconsciousness (%) | 2.2 | 2.4 | 5.3 | 0.011 | 2.7 | 3.6 | 8.6 | <0.001 |
| Fever (>=37.5℃) (%) | 71.2 | 79.7 | 83.4 | <0.001 | 78.6 | 79.6 | 80.1 | 0.822 |
| Cough (%) | 53.9 | 52.0 | 63.4 | <0.001 | 56.6 | 55.2 | 69.2 | <0.001 |
| Sputum (%) | 21.0 | 20.5 | 26.4 | 0.039 | 21.7 | 21.4 | 34.1 | <0.001 |
| Sore throat (%) | 25.9 | 22.4 | 21.2 | 0.138 | 25.1 | 20.0 | 16.1 | 0.002 |
| Rhinorrhea (%) | 17.8 | 11.6 | 12.7 | 0.018 | 15.4 | 10.0 | 11.6 | 0.074 |
| Dysgeusia (%) | 20.6 | 14.3 | 14.9 | 0.017 | 18.1 | 10.9 | 12.0 | 0.007 |
| Olfactory disorder (%) | 19.7 | 11.3 | 11.2 | <0.001 | 15.9 | 6.5 | 8.1 | <0.001 |
| Shortness of breath (%) | 16.4 | 22.8 | 45.1 | <0.001 | 26.1 | 30.7 | 57.2 | <0.001 |
| Abdominal pain (%) | 2.6 | 0.5 | 3.2 | 0.097 | 2.7 | 2.9 | 2.2 | 0.819 |
| Abdominal distension (%) | 0.2 | 0.0 | 1.2 | 0.058 | 0.6 | 0.0 | 1.2 | 0.307 |
| Hematochezia (%) | 0.6 | 0.0 | 0.5 | 0.544 | 0.5 | 0.0 | 0.6 | 0.660 |
| Diarrhea (%) | 15.8 | 15.6 | 16.6 | 0.903 | 14.5 | 24.6 | 16.3 | 0.009 |
| Nausea (%) | 7.7 | 5.4 | 9.2 | 0.179 | 7.8 | 13.0 | 7.5 | 0.099 |
| Fatigue (%) | 38.1 | 47.1 | 55.9 | <0.001 | 43.2 | 57.6 | 65.2 | <0.001 |
| Data show mean±standard deviation or percentage values. Data were analyzed using the χ^2^ test or by analysis of variance where appropriate.  BMI, body mass index; COPD, chronic obstructive pulmonary disease; CXR, chest X-ray; GGO, ground-glass opacity | | | | | | | | |

| **Supplementary Table 3**. Clinical characteristics according to whether two CXRs were performed within 48 hours | | | |
| --- | --- | --- | --- |
| Parameters | Patients with two CXR within 48 hours | Patients without two CXR within 48 hours | P value |
|  |  |  |  |
|  | (n = 1656) | (n = 269) |  |
| **Demographics** |  |  |  |
| Age, years | 59.5 (±17.6) | 55.0 (±19.1) | <0.001 |
| Sex, female/male (%) | 32.5/67.5 | 42.8/57.2 | 0.001 |
| BMI, kg/m^2^ | 24.6 (±4.8) | 24.2 (±4.5) | 0.291 |
| Current or previous smoker (%) | 46.8 | 46.3 | 0.884 |
| Brinkman index | 660.8 (±803.3) | 868.5 (±1338.4) | 0.032 |
| Number of days from symptom onset to hospitalisation, days | 6.0 (±4.6) | 6.1 (±4.1) | 0.802 |
| **Comorbidities** |  |  |  |
| Hypertension (%) | 37.1 | 31.3 | 0.073 |
| Diabetes mellitus (%) | 23.3 | 22.9 | 0.897 |
| Cardiovascular disease (%) | 10.8 | 7.3 | 0.078 |
| Malignancy (%) | 6.9 | 6.9 | 0.996 |
| Autoimmune disease (%) | 3.7 | 2.3 | 0.252 |
| COPD (%) | 5.0 | 3.9 | 0.441 |
| Asthma (%) | 6.8 | 5.8 | 0.535 |
| Hyperuricemia (%) | 11.1 | 8.1 | 0.151 |
| Chronic liver disease (%) | 3.3 | 3.1 | 0.846 |
| Chronic kidney disease (%) | 8.0 | 6.3 | 0.349 |
| **Severity** |  |  | 0.003 |
| Asymptomatic (%) | 3.5 | 2.6 |  |
| Mild (%) | 51.0 | 63.2 |  |
| Severe (%) | 29.8 | 22.7 |  |
| Most severe (%) | 15.6 | 11.5 |  |
| Data show mean±standard deviation or percentage. Data were analyzed using the χ^2^ test or t test, where appropriate.  BMI, body mass index; COPD, chronic obstructive pulmonary disease; CXR, chest X-ray | | | |

| **Supplementary Table 4.** Clinical features of the patients | | | | |
| --- | --- | --- | --- | --- |
| Parameters | All | Patients showing no deterioration in CXR findings | Patients showing rapid deterioration in CXR findings | P value |
|  |  |  |  |  |
|  | (n = 1656) | (n = 1488, 89.9%) | (n = 168, 10.1%) |  |
| **Vital signs** |  |  |  |  |
| Body temperature, ℃ | 37.3 (±0.9) | 37.2 (±0.9) | 37.8 (±1.1) | <0.001 |
| Systolic blood pressure, mmHg | 129.4 (±20.3) | 129.3 (±20.1) | 130.3 (±22.5) | 0.555 |
| Diastolic blood pressure, mmHg | 80.4 (±13.6) | 80.4 (±13.4) | 80.2 (±14.9) | 0.905 |
| Heart rate, beats/min | 87.2 (±16.8) | 86.5 (±16.4) | 92.8 (±19.6) | <0.001 |
| Respiratory rate, breaths/min | 19.5 (±4.7) | 19.3 (±4.7) | 21.3 (±5.3) | <0.001 |
| SpO_2_ % | 95.8 (±3.3) | 96.0 (±3.2) | 94.5 (±4.1) | <0.001 |
| Oxygen support (%) | 23.2 | 21.0 | 45.2 | <0.001 |
| **Laboratory findings** |  |  |  |  |
| WBC, ×10^3^/μL | 5.86 (±3.01) | 5.81 (±2.96) | 6.30 (±3.39) | 0.048 |
| Neutrophils (%) | 69.9 (±13.3) | 69.2 (±13.6) | 77.0 (±10.9) | <0.001 |
| Lymphocytes (%) | 21.4 (±12.1) | 22.0 (±12.4) | 15.9 (±8.5) | <0.001 |
| Hb, g/dL | 14.0 (±1.9) | 14.0 (±1.9) | 13.9 (±2.1) | 0.250 |
| Plt, ×10^4^/μL | 19.9 (±7.8) | 20.1 (±7.8) | 17.8 (±7.7) | <0.001 |
| Alb, g/dL | 3.7 (±0.6) | 3.7 (±0.6) | 3.4 (±0.6) | <0.001 |
| AST, IU/L | 42.9 (±73.4) | 42.1 (±76.1) | 49.6 (±43.2) | 0.211 |
| ALT, IU/L | 40.4 (±94.1) | 40.1 (±98.6) | 42.4 (±34.5) | 0.768 |
| BUN, mg/dL | 17.9 (±15.0) | 17.4 (±15.0) | 21.7 (±14.7) | <0.001 |
| Cre, mg/dL | 1.11 (±1.52) | 1.08 (±1.45) | 1.42 (±2.01) | 0.006 |
| LDH, IU/L | 279.0 (±144.2) | 271.1 (±144.2) | 348.6 (±144.6) | <0.001 |
| Na, mEq/L | 138.2 (±3.8) | 138.4 (±3.8) | 137.1 (±4.1) | <0.001 |
| K, mEq/L | 3.99 (±0.5) | 3.99 (±0.5) | 4.04 (±0.5) | 0.1627 |
| BNP, pg/mL | 76.6 (±411.9) | 60.8 (±208.3) | 162.7 (±928.7) | 0.018 |
| Ferritin, ng/mL | 566.3 (±610.5) | 533.4 (±588.0) | 836.6 (±772.1) | <0.001 |
| KL-6, U/mL | 333.5 (±332.4) | 329.3 (±331.8) | 366.8 (±337.2) | 0.210 |
| HbA1c, % | 6.4 (±1.3) | 6.4 (±1.3) | 6.8 (±1.5) | <0.001 |
| Fibrinogen, mg/dL | 487.6 (±153.1) | 481.2 (±154.1) | 539.2 (±144.7) | <0.001 |
| D-dimer, μg/mL | 2.7 (±9.7) | 2.4 (±9.0) | 4.9 (±14.2) | 0.003 |
| CRP, mg/dL | 5.1 (±6.1) | 4.7 (±5.9) | 8.9 (±7.4) | <0.001 |
| **Radiological findings** |  |  |  |  |
| CXR GGO (%) | 68.7 | 65.6 | 95.2 | <0.001 |
| Bilateral (%) | 55.7 | 52.0 | 86.9 |  |
| Unilateral (%) | 13.0 | 13.6 | 8.3 |  |
| CXR consolidation (%) | 29.9 | 26.9 | 55.1 | <0.001 |
| Bilateral (%) | 21.0 | 18.2 | 44.9 |  |
| Unilateral (%) | 8.9 | 8.7 | 10.2 |  |
| Data show mean±standard deviation or percentage. Data were analyzed using the χ^2^ test or t test, where appropriate. Alb, albumin; ALT, alanine aminotransferase; AST, aspartate aminotransferase; BNP, brain natriuretic peptide; BUN, blood urea nitrogen; Cre, creatinine; CRP, C-reactive protein; CXR, chest X-ray; GGO, ground-glass opacity; Hb, hemoglobin; K, potassium; KL-6, Krebs-von-den-Lungen-6; LDH, lactate dehydrogenase; Na, sodium; Plt, platelet; SpO_2_, saturation of percutaneous oxygen; WBC, white blood cell | | | | |

| **Supplementary Table 5.** Effect of radiographic findings on rapid deterioration in CXR findings | | | | |
| --- | --- | --- | --- | --- |
| Modality | Variable | aOR | 95% CI | P value |
| CXR | CXR bilateral GGO | 9.00 | 4.32-18.8 | <0.001 |
|  | CXR unilateral GGO | 4.39 | 1.79-10.7 | 0.001 |
|  | CXR bilateral consolidation | 2.43 | 1.68-3.06 | <0.001 |
|  | CXR unilateral consolidation | 1.72 | 0.96-3.06 | 0.066 |
| The adjusted odds ratio was estimated by logistic regression. 95% CI, 95% confidence interval; aOR, adjusted odds ratio; CXR, chest X-ray; GGO, ground-glass opacity | | | | |
